# Exploring the relationships between the gut microbiome composition and movement patterns of laying hens in a multitier cage-free housing system

**DOI:** 10.1371/journal.pone.0340059

**Published:** 2026-01-09

**Authors:** Anaïs Cazals, Sabine Gebhardt-Henrich, Quentin Berger, Marie-Noëlle Rossignol, Deborah Jardet, Michael J. Toscano, Tatiana Zerjal

**Affiliations:** 1 Université Paris-Saclay, INRAE, AgroParisTech, GABI, Jouy-en-Josas, France; 2 Center for Proper Housing: Poultry and Rabbits, Division of Animal Welfare, VPHI, University of Bern, Zollikofen, Switzerland; Tokat Gaziosmanpaşa University: Tokat Gaziosmanpasa Universitesi, TÜRKIYE

## Abstract

In this study we investigated the relation between caecal microbiota composition and movement patterns in laying hens. We used hens from Pure line matings of Hendrix Genetics to continuously monitor the movement of individuals in a connected three-tier aviary throughout the laying period, from 18 to 60 weeks of age. The aviary contained three vertical tiers: a top-level, mid-level, and lower-level. In addition, the aviary had a floor littered and an attached wintergarden which was accessible from approximately 21 WOA onwards. Differences in the hens’ use of space were observed including: differences in the number of visits and time spent in the wintergarden and litter areas. Microbiota characterization, using 16S rRNA gene sequencing from 237 samples, revealed an association (P < 0.05) between microbiome composition and the number of visits to the litter. We observed differences (adjusted P-value < 0.05) between hens that frequently visited the litter (>30 times/day) and those that visited the litter less often (<10 times/day) in five bacterial families and seven genera. Notably, hens classified as visiting the litter less often, showed an increased abundance of *Coriobacteriales*, *Peptococcales*, *Oribacterium* and *Lachnoclostridium* taxa. Overall, this study offers new insights on the potential role of the microbiota in hen movement patterns.

## Introduction

Laying hens in cage free housing systems exhibit a wide range of behaviors that appear to be strongly consistent within individuals [1–5] and can be used as indicators of welfare [6]. By understanding individual behavioral differences, we can enhance welfare in farming systems [6] through improved management [7] and breeding strategies [8,9]. Individual differences can be influenced by environmental factors (e.g., space availability, temperature), intrinsic motivations (feeding or laying motivation), the presence of conspecifics (e.g., social interactions), and health status [10–16].

More recently, growing evidence supports also an association between an animal’s microbiota and behavioral patterns [17], highlighting the role of the gut-brain axis in brain functions [6,18,19]. Studies in quail [20] and poultry [21] show that changes or absence of gut microbiota can alter emotional reactivity, stress responses, and social behaviors. Environmental factors such as rearing systems [10] and ranging patterns [22] also influence both microbial composition and behavior. Overall, these findings support the existence of a microbiota–gut–brain axis in avian species, suggesting that gut microorganisms play a key role in shaping stress regulation and social interactions [23].

Within cage-free aviary housing systems, hens can access multiple resources including: litter, perches, feeders, external areas, and nestboxes intended for egg-laying. Individual hens can display a wide range of movement and location patterns that result from their use of these areas [24]. The expression of these patterns raises important questions about the underlying mechanisms that drive individual behavioral variability including how these associations develop and influence other traits of interest, such as health. In this study we investigated whether variation in gut microbiota composition could explain part of this individual behavioral variability. To do so we tracked the location of hens housed in a cage-free aviary housing system and: (i) characterized the diversity and composition of the caecal microbiota from individual hens, (ii) compared microbiota profiles between hens displaying extreme behavioral patterns in the use of the aviary space, and (iii) identified microbial taxa potentially associated with specific hen’s movement profile. As our study was operating with minimal a priori information, we considered our effort to be exploratory with the broad hypotheses that differences in hens’ use of the litter and wintergarden would lead to differences in microbial diversity and composition.

## Materials and methods

### Animals and housing

The animals used in this study have been described previously, along with their rearing conditions [25]. The same flock was also used to validate the tracking technology employed in our study [26]. In brief, four thousand eight hundred chicks were provided by Hendrix Genetics (5831 CK Boxmeer, The Netherlands). The chicks were specialized white-colored parental crosses from Pure Line matings using 100 sires. The chicks arrived on the site of Aviforum (Zollikofen, Switzerland) at one-day of age. Wing tags indicating the sire were applied at the hatchery during standard processing. The chicks were placed into eight rearing pens, with equal numbers of sires represented in each pen. Four pens of the rearing barn contained the Landmeco Harmony rearing aviary (Landmeco A/S, Olgod, Denmark). The total space allocation was 42.74 m^2^ in pens 1–3, and 41.61m^2^ in pen 4, with total pen dimension of 4.80 m × 3.50 m, and a wintergarden of 4.90 m × 2.55. The other half of the barn contained the Inauen Natura aviary (R. Inauen AG, Appenzell, Switzerland), with total space allocation of 40.99 m^2^, pen dimension of 4.90 m × 4.55, and a wintergarden of 4.95 m × 3.45 m. Pens of both aviary types were arranged in series, interconnected, and exposed to the same environmental conditions (e.g., temperature, airflow). Beyond the housing differences, all management protocols (e.g., feeding, lighting schedule) were the same. At approximately 15 weeks of age (WOA), 1,125 pullets from 25 of the 100 sires were fitted with a passive RFID tag (125 kHz) and a pen-specific color leg band. The RFID fitting procedure was done in the rearing barn to not delay transfer of hens to the on-site laying barn, which needs to occur within a fixed, six-hour window. The allocation of leg bands was done in a manner to ensure that the five pens to be used in the laying barn were stratified for sire and rearing pen resulting in approximately 40 hens per sire across the five laying pens. At 18 WOA, the hens were transported to the laying barn at the same site, containing a Bolegg Terrace aviary (Vencomatic Group, 5521 DW Eersel, The Netherlands) divided into 20 replicated pens with each containing 225 hens. The five pens used for this study were all adjacent. The aviary system was a three-tiered aviary consisting of: a top-level tier (TLT), nest box tier (NBT), and lower-level tier (LLT). In addition, the aviary had a littered floor area (LIT) and attached wintergarden (WG) area which was accessible from approximately 21 WOA onwards. The aviary had been modified to Swiss conditions so that the drinker, normally at the NBT, was moved to the TLT. Resources were provided on the different aviary tiers: feeding chains and nipple drinkers on the lower tier provided food and water; group nests provided on the middle tier. Food, water, and perches (diameter: 3.2 cm, length: 230 cm) were available on the top tier. Additional perches were installed on both sides of the aviary structure to facilitate movements between tiers. Animal density was 7.4 hens/ m^2^ of accessible area (including all grid areas of the lower and upper tiers and littered floor area). The nestboxes ran the length of the pen and were divided into two units per side resulting in a total length of 6.6 m. The WG provided an additional 9.32 m^2^ and contained wood shavings and a dustbathing area filled with sand.

Feeding, vaccination, duration of light, and other management procedures followed common guidelines and instructions for the Dekalb hybrid [27]. The maximum length of light was 14 h from 03:00–17:00 and the natural light through windows was supplemented by artificial light, whereas access to the WG was between 10:00 and 16:00. A staff member would ensure all hens returned to the barn’s interior. Litter was replenished every 30–60 days depending on season and litter quality, but all pens had their litter removed at the same time. When fresh litter was provided, it was given as a block and hens then distributed it themselves. The experiment was approved by the Veterinary Office of the Canton of Bern (BE4/2021, approved on Sept. 1, 2021) and met all cantonal and federal regulations for the ethical treatment of laboratory animals.

### Tracking data

To record and monitor the duration of time and frequency of passage between the earlier defined aviary zones (i.e., TLT, NBT, LLT, LIT, and WG), we used the Gantner Pigeon Systems GmbH (6780 Schruns, Austria). Hens were equipped with leg-mounted transponders, which were registered when they came within 10 cm of an antenna. Thirty-two 12-field SPEED antennas (75 × 35 cm) per pen were distributed as described in [25]. Tracking of the birds began five days (d) after arrival in the laying barn to ensure that the hens were well acclimated and continued for 290 d (until 59 WOA) from the first day of arrival in the aviary. Animals that died during the study (14 birds) or lost their RFID tags (four birds) were removed from the database. Furthermore, 68 d of unusual disturbances (e.g., health assessments for another project in the barn using these or other pens that occurred in 14 day blocks spread over the study period) were deleted. Health assessments involved a large number of people entering the barn and collecting all hens in the pens, which we believed disturbed the entire barn. Movement records were collected for each animal based on the number of daily visits to that area (i.e., when a hen-specific transponder registered at an antenna within a zone) and time spent in each zone. The time spent in minutes in a particular zone was summed for each animal on each day in the barn. In total, 1,106 animals with 937,740 cumulative daily movement records were retained for further analyses. From this overall dataset, as our primary objective was to investigate potential effects of wintergarden use on the gut microbiota, we preferentially selected birds showing either very low or very high WG usage in the four weeks prior to collection, i.e., 50–55 WOA. Of this subgroup, 37 birds never entered the WG, 25 had a relatively low usage of the WG, and 62 birds had relatively high usage of the wintergarden, i.e., 62 hens with none or low WG usage and 62 with high usage. To achieve a balanced representation of approximately 50 birds per pen, an additional 119 birds were included, resulting in a total sample of 243 birds. These additional birds were randomly selected and were not specifically chosen based on WG use. Although our main selection criteria was WG use, we also considered the use of the LIT area within our analysis, given that litter (available in both the LIT and WG) and external access (i.e., WG only) may lead to an increased exposure to pathogens [28,29]. The provision of litter also allows birds to perform dustbathing and foraging, behaviors they are highly motivated to perform and indicate welfare [30].

### Caecal content collection, DNA extraction and 16S rRNA gene sequencing

Caecal content was collected from the 243 focal birds at 60 WOA. Immediately after euthanasia, the abdominal cavity was opened, both caeca were removed, and their contents were pressed out into a sterile petri dish. The content was then homogenized using a single-use getable plastic stirrer and a subsample was transferred into a sterile 2 ml screw-top cryotube. Samples were immediately frozen in liquid nitrogen, and stored at −80 °C until laboratory analysis. DNA extraction and 16S rRNA gene sequencing were performed at the @bridge platform (INRAE, Jouy-en-Josas, France). Individual caecal DNA was extracted from an average 200 mg of frozen caecal content according to the platform’s procedure (see S1 File). DNA concentration and quality (A260/280 ratio) were assessed by fluorometric quantification (Qubit, Thermofisher Scientific, USA) and DNA samples were stored at −20°C. Only the DNA samples with a A260/280 ration between 1.8 and 2.1 and a concentration of at least 25 ng/µl were used. Amplification of the V3-V4 hyper-variable region of the 16S rRNA gene was performed with two rounds of PCR with universal V3-V4 primers (S1 File). Negative controls to assess technical background were included using nuclease-free water (Qiagen, Germany) in place of the extracted DNA during the library preparation. Sequencing of amplicons was carried out using Illumina MiSeq technology (2 x 250 paired-end reads) following the standard protocol.

### Bioinformatics analysis

We used FastQC to control quality of each raw fastq file. Filtering, trimming, and chimera removal were applied to the reads, and the construction of the amplicon sequence variants (ASV) table was performed using the DADA2 v1.32.0 package [31] in R software (version 4.4.1), following the authors’ recommendations. Taxonomic annotation was performed using the Silva Dataset v132 [32]. For quality control, ASVs with a relative abundance below 0.5% per sample and samples with fewer than 10,000 reads (n = 3) were excluded from the analysis. While we are aware of the potential disadvantages of rarefaction, such as data loss and reduced statistical power, we applied it to standardize sequencing depth across samples, a common practice that facilitates comparison of alpha and beta diversity metrics. The final abundance table was then rarefied to 17,574 counts per sample, resulting in 957 ASVs across 240 samples.

### Statistical analysis

From the 243 DNA samples, we obtained 240 samples with valid microbiota data (i.e., more than 10,000 sequencing reads). Then, three were excluded due to incomplete tracking data (missing data, see S1 Table); further analyses were then conducted on 237 samples. The traits analyzed included the median number of daily visits to the LIT (median_LIT_visits), the median duration of time spent in the LIT per day (median_duration_LIT), and the median duration of time spent in the WG per day (median_duration_WG). To define the extreme groups with contrasting movement for each trait, we selected the top 20% of individuals with the highest and lowest values and assigned them to their respective classes. For median_duration_WG, the low class included individuals who did not visit the WG (i.e., duration = 0). The subset of samples was distributed across all pens, as described in S2 File.

The packages phyloseq v1.48.0 [33] and vegan v2.6-8 [34] were used to perform diversity analyses on normalized data. To estimate within individual microbiota richness and evenness as well as the microbiota diversity between samples, alpha diversity and beta diversity were measured using the Shannon and Whittaker indices, respectively. ANOVA tests were performed to assess the differences in alpha- and beta-diversity between classes of hens, with pen included as a fixed effect. We note that the sire was found to be statistically insignificant for all traits and was not included in the model. Bray-Curtis distances were calculated and plotted using a non-metric multidimensional scaling (NMDS) representation. After removing missing data (NA), Permutational Multivariate Analysis of Variance (PERMANOVA) was performed using the *adonis2* function with 9,999 permutations to assess the significance of movement classes and pen as fixed effect on ASV abundances. The DESeq2 version 1.44 package [35] was used to identify differentially abundant (DA) ASVs between classes of hens. ASVs were subsequently aggregated at different taxonomic levels to identify differentially abundant orders, families, and genera.

To analyze the correlation between movement classes, we used a Spearman correlation test due to the non-normality of the traits.

The metadata table and the ASV table are available in supplementary tables S1 and S2 Tables, respectively.

## Results

### Distribution of movement classes

**Fig 1:**
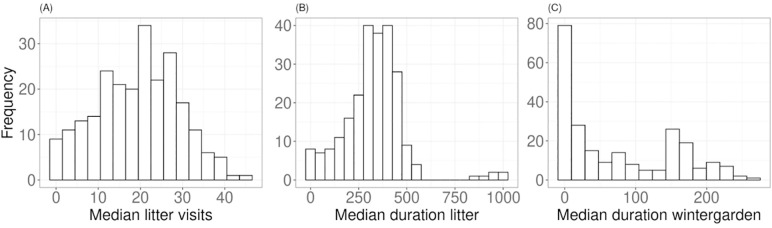
Distribution of movement traits. For the 237 samples, median number of daily visits to the LIT **(A)**, median duration of time spent in the LIT per day in minutes **(B)**, and median duration of time spent in the WG per day in minutes **(C)**.

In the 237 samples analyzed in this study, the median LIT visits, median duration in LIT, and median duration in WG have a mean value of 19.32 visits, 327.91 minutes, and 74.53 minutes, respectively, with a standard deviation of 9.78, 159.44 and 77.32 (Fig 1). As a reminder, 124 birds were initially selected according to their WG usage (either very low or very high), which could explain the observed results, namely the lack of a normal distribution (Fig 1, right panel). Without pre-selection, the mean of the median duration in WG was 54.04 min with a standard deviation of 60.19.

The Spearman correlation test revealed a moderate correlation between “median LIT visit” and “median duration LIT” (P-value < 0.01; Rank correlation coefficient (Rho) = 0.64), as well as between “median LIT visit” and “median duration WG” (P-value < 0.01; Rho = 0.60). However, no correlation was found between “median duration LIT” and “median duration WG” (P-value = 0.14; Rho = 0.10).

Extreme groups with contrasting movement patterns for each movement traits were defined and described in Table 1.

**Table 1:** Summary of movement trait variables in movement classes.

	Low				High			
		Nb	Mean	SD		Nb	Mean	SD
**median_LIT_visits (number of times)**	LIT-	47	5.30	±3.19	LIT+	47	33.40	±4.05
**median_duration_LIT (min)**	MDL-	47	115	±71	MDL+	47	528	±164
**median_duration_WG (min)**	MDW-	33	0	±0	MDW+	47	193	±28

Number of samples (Nb), Mean, and Standard Deviation (SD) for each of the three quantitative variables analyzed, in the global population and in the extreme high and low groups.

### Global structure and diversity of bacterial communities

**Fig 2:**
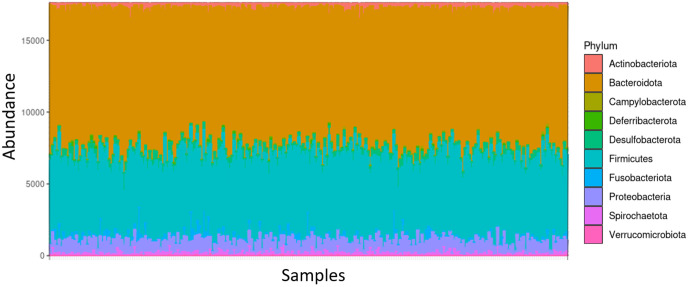
Microbiome composition at the phylum level. Abundance of bacterial phyla (reads) after rarefaction at 17,574 reads for the 240 samples.

From the 957 ASV, the taxonomic affiliation allowed the identification of 10 phyla, 28 orders, 44 families, and 96 genera. At the phylum level, the bacterial community was dominated by *Bacteroidota* and *Firmicutes*, followed by *Proteobacteria* (Fig 2). The mean alpha diversity, measured by the Shannon index, was 5.33 (± 0.15) and the beta diversity, assessed as 0.30 (± 0.036).

A pen effect was found on alpha diversity (P-value < 0.01; S3 File) but not on beta-diversity (P-value > 0.1; S3 File). Moreover, the PERMANOVA test highlighted an impact of pen on the overall gut microbiome composition (P-value < 0.01).

### Analysis of the mean litter visit trait

**Fig 3:**
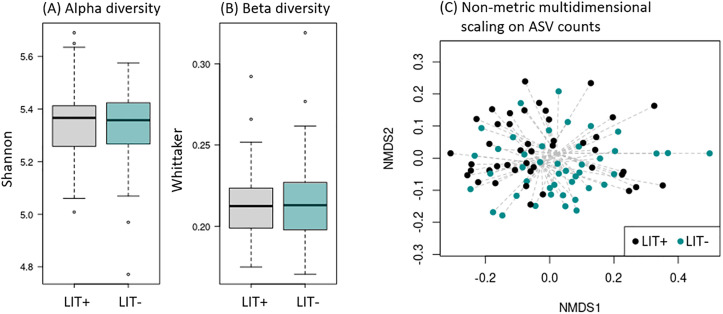
Comparison of gut microbiome between extremes of median LIT visits (LIT+ and LIT- groups). **A and B:** Alpha- (Shannon index) and Beta- (Whittaker index) diversity after correction for pen. **C:** Non-metric multidimensional scaling representations of the ASV abundance of caecal microbiome using Bray-Curtis dissimilarity.

For median_LIT_visits, hens were classified into two groups: those with fewer than 10 daily visits (LIT-, bottom 20%) and those with more than 30 daily visits (LIT + , top 20%). Analysis of alpha and beta diversity using the Shannon and Whittaker indices, respectively, showed no differences between groups (Fig 3A, 3B and S4 File). However, the PERMANOVA analysis, adjusted for pen, revealed an impact of LIT visit frequency on gut microbiome composition (P-value < 0.05), although clustering between groups was not evident in the NMDS plot (Fig 3C).

Among the 957 identified ASVs, nine showed significant differential abundance (Padj < 0.05, log-fold changes between 0.02 and 1) between the median litter visit classes, with six ASVs enriched in the LIT+ group and three ASVs in the LIT- group (Table 2 and S3 Table). At higher taxonomic levels, seven genera, five families, and three orders were differentially abundant between groups (Table 1 and S3 Table).

**Table 2:** Differentially abundant ASVs, orders, families, and genera between LIT+ and LIT- groups.

	ASV	Order	Family	Genus
**LIT+**	ASV837 ASV125 ASV228 ASV306 ASV937 ASV417	*Flavobacteriales*		Pygmaiobacter
**LIT-**	ASV369 ASV233 ASV32	*Coriobacteriales* *Peptococcales*	*Coriobacteriales Incertae Sedis* *Coriobacteriaceae* *Atopobiaceae* *Eggerthellaceae* *Peptococcaceae*	Lachnoclostridium Enorma Oribacterium Olsenella CHKCI002 Peptococcus

### Analysis of other movement traits

Similar analyses were conducted for the two other behavioral traits, median_duration_LIT and median_duration_WG. Comparison of classes for each trait revealed no differences in alpha- and beta-diversity, nor in the PERMANOVA analysis (P-value > 0.05; S5 File). In addition, no ASV, order, family, or genus were identified differentially abundant between the extreme groups.

## Discussion

Although the analyzed hens had a fairly homogenous caecal microbiome, our study identified unique microbial variants that related to the pen within the barn as well as individual movement characteristics. We believe that these findings are the first to demonstrate such differences in laying hens occupying the barn based on movement profiles.

### Microbiota diversity and composition in free-cage housing system

Despite movement pattern variability among hens, our results revealed a global homogeneous microbiota structure, both in terms of alpha and beta diversity. The mean Shannon index (5.33 ± 0.15) is consistent with previous findings in caecal content of adult laying hens in similar husbandry conditions [22,36,37]. The relative low beta-diversity values observed (0.30 ± 0.036) support the notion of a relatively uniform microbial community across individuals, sharing similar environments, diets, developmental stages or sex [38].

Although the microbiota of individual hens was fairly homogenous, we found that the pen environment influenced microbiota composition, particularly in terms of alpha diversity (P-value < 0.01) and global community structure (PERMANOVA, P-value < 0.01). The differences between pens highlight the role of micro-environmental factors in affecting microbiota composition. Even in our pens with identical genetics, diet, management, and housing, pre-existing individual-level differences in behavior, shaped by genetics, early experiences, or epigenetics, can lead hens to explore, interact, and inhabit space differently [39]. These behavioral differences, likely of a stochastic, dynamic nature, could influence micro-environmental exposure (e.g., to microbes on surfaces, litter or feeders), resulting in distinct microbiota composition even across otherwise uniform pens. Indeed, recent studies indicate that the housing environment itself (air, dust, litter, and other barn matrices) can serve as a substantial source of microorganisms influencing avian microbiota [40–42]. The spread of distinct microbial populations within the pen would be reinforced by coprophagy which has been previously documented in poultry [38].

As previous studies indicate, environmental microbial populations can actively shape gut colonization. A limitation of this study is that we did not directly analyze the microbiota of the pen environment; nevertheless, we included pen as a fixed effect in the statistical analysis to specifically account for possible pen differences. Another important point is that, although our research aviary is smaller than a typical commercial facility, commercial barns are often divided into distinct adjacent pens similar to those used in this study, although housing several thousand animals. Therefore, we believe the observed differences we observe remain commercially relevant.

### Link between gut microbiota and movement traits

The range of studies in poultry linking gut microbial composition and behavior traits is rather wide, including feather pecking, other injurious behaviors, fear responses to humans, housing system (cage vs. free-range), or general activity patterns (outdoor vs. indoor-preferring) [10,43–46]. In our study, we investigated in depth the relationship between the gut microbiota and hen daily routine movement patterns within a single housing system. Among the three movement traits analyzed and their associated area of focus, only the frequency of visits to the litter area was associated with differences in gut microbiota composition. When designing the study and selection criteria, the WG use seemed the most promising factor due to marked differences in its use among hens, the direct exposure to external factors (e.g., wind, pollen), and the potential for hens to encounter distinct bacterial communities not present in other parts of the aviary. However, use of the WG did not reveal any associations with the microbiome. While the moderate correlation between the median number of LIT visits and durations spent in the LIT or WG might be expected to result in a similar association with the microbiota among the three analyzed traits, this was not the case. The lack of association of WG and LIT duration with microbial populations suggests that it is specifically the frequency of moving to and from the litter that is associated with microbiome populations.

Although our study was not designed or intended to establish causal relationships, there are known associations observed in poultry and mice between movement and specific microbiome populations, which support our findings. Chen et al. (2019) showed that different rearing systems (cage vs. free-range) affect not only chicken behavior but also gut microbiome diversity and community structure [10]. Sztandarski et al. (2022) showed that chickens’ ranging preferences (outdoor, moderate, indoor) influenced gut microbial activity (enzymes activities, SCFA concentrations) and the abundance of key bacterial groups, linking daily movement patterns to microbiota composition. In mice, a link between motivation for exercise and microbiota was recently described [47], where microbiome-dependent endocannabinoid metabolites produced in the gut increased dopamine levels in the brain during exercise, providing potential mechanistic insights into how gut microbiota may influence behavior.

### Stress-associated gut microbial signatures in divergent behavior

Although further studies are required to explore whether gut-derived compounds could directly influence hen behavior or vice versa, the current study did identify specific microbial populations associated with litter visits, a profile we previously linked with stressful conditions detailed below. Furthermore, the specific microbiome populations identified in the current study were previously linked with stressful conditions in various species, strengthening the position for a causal relationship. We believe this is the most exciting aspect of our findings as it suggests an association between stress, movement behavior, and specific microbiome populations. These associations remain interpretative, but are discussed in light of the large body of literature supporting links between similar microbial taxa and stress-related conditions.

Our group has previously shown that fractures to the keel bone, an injury believed to cause pain [48], a depressive state [49], and widely affecting laying hens (i.e., upwards of 80% of the flock [50–52]), is associated with reduced daily transitions between tiers [12] and more time in the TLT [53]. Within the same vein, birds characterized as less aggressive (Grethen et al., submitted) or observed with toe injuries (Gomez et al., 2024) spent more time in the TLT, had reduced transitions between tiers, and spent less time in the litter [54]. Although these health characterizations were not made in hens of the current flock [54], they were made in the same barn and support that greater time in the TLT with less transitions to the litter is associated with birds that are injured and likely experiencing a negative affective state. We have previously suggested that birds could be adopting this profile in order to facilitate recovery [12].

It is also been demonstrated in the literature that the specific microbial populations linked with litter visit class in the current study may be stress related. Hens classified in the LIT- group (less than 10 daily litter visits) demonstrated an increased abundance of the *Coriobacteriales* order. Elevated levels of *Coriobacteriales* have been observed in mice exposed to stress factors (immobilization, or exposure to predator odors) [55], as well as humans experiencing stress, such as tunnel workers or patients with depressive episodes and bipolar disorder [56,57]. Our study identified a notable presence of *Coriobacteriales* among the DA taxa between LIT+ and LIT- groups, including three ASVs (ASV369, ASV233, ASV32) and multiple genera and families (Enorma, Olsenella, CHKCI002; Coriobacteriales Incertae Sedis, Coriobacteriaceae, Atopobiaceae, and Eggerthellaceae) more abundant in the LIT- hens. In poultry, recent studies have shown that an increase in *Coriobacteriales* is associated with cold stress [58], and an increase in the Eggerthellaceae family and the CHKCI002 genus within this family has been linked to heat stress [59].

In addition, individuals classified within the LIT- group exhibited a higher abundance of *Peptococcales* (one bacterial genus, a family, and the overall order), a bacteria associated with stressful conditions. A study conducted in rats demonstrated that psychological stress could modulate the expression of tight junction proteins in the intestinal barrier, a modulation positively correlated with the abundance of *norank_f_Peptococcaceae*, a member of the *Peptococcales* order [60]. Tight junction proteins, such as claudin-5, occludin, and ZO-1, are crucial for maintaining intestinal barrier integrity, and their dysregulation has been implicated in various stress-related pathologies. Focusing on commercial poultry, a study in broilers revealed that during thermal stress (an increase of 10°C), the abundance of the *Peptococcaceae* family in ceacal contents increased [61].

The genus *Oribacterium* was found to be more abundant in the LIT- group, a finding that aligns with a study in humans that analyzed the salivary microbiome in relation to anxiety and reported increased *Oribacterium asaccharolyticum* species abundance [62].

The genus *Lachnoclostridium* was found to be more abundant in the LIT- group. Interestingly, a study on stress-resistant mice reported lower abundance of this genus [63], aligning with our findings. In that study, stress-induced abnormalities in the gut microbiota were shown to disrupt microglia-neuron interactions in the hippocampus. Furthermore, fecal microbiota transplants from stress-resistant mice, characterized by a distinct microbial composition (including lower levels of *Lachnoclostridium*), were found to mitigate the effects of stress in recipient animals. Similarly, *Lachnoclostridium* abundance were enriched in mice with depressive-like behaviors [64].

Finally, the genus *Oscillibacter* (ASV306) was found to be more abundant in the LIT+ group. Interestingly, a study in chickens reported an increase of this genus in a genetic line exhibiting lower aggressive behavior [65]. Physiologically, this line showed higher levels of serotonin and tryptophan in the brain, along with lower corticosterone levels and heterophil-to-lymphocyte ratios, indicating a reduced stress response. In another study in chicken, *Oscillibacter* was more abundant in the control group compared with the cold stress group [58]. Moreover, they found also the genus *Tyzzerella* more abundant in the control group compared with the cold stress group, which aligns with our findings that ASV937 (*Tyzzerella)* was more abundant in LIT + group [58].These findings suggest that *Oscillibacter* and *Tyzzerella* may be reduced in animals suffering a stressful condition.

Taken together, these findings support the hypothesis, acknowledging that it remis speculative, that hens making fewer litter visits may experience higher stress levels. Future studies should include specific measurements of stress related indicators to explore further this hypothesis.

### Does movement, social interaction or health status link with microbiota?

The apparent association between behavior, microbial populations, and stress raises important questions regarding directionality: are behavioral differences shaping microbiota for example, or are pre-existing microbiota differences influencing behavioral and stress-related phenotypes?

In cage-free housing, birds can move within barn areas that differ in their structure and environmental factors (e.g., temperature, ammonia). Recent work by our group [1,2] and others [3–5] has provided evidence of inter-individual differences in the use of these areas, as well as individual variation in their use over time [24]. In parallel, there is also evidence that birds may move together in subgroups [13], although the strength and nature of association may fluctuate with time [14] and be affected by their health status.

The substrates present in specific areas of the multitier aviary may harbor different microbial ecosystems that could influence gut microbiome populations. If individual birds used the litter areas in a different manner (e.g., frequency and/or duration), this could explain how the usage-specific microbiome populations developed. In our study, the association between microbiota and movement patterns was observed with the frequency of visits to the litter, but not with the time spent on the litter, which suggests that factors other than the microorganisms present in the litter may influence the microbiota composition of the gut. For instance, birds with reduced visits but similar durations may engage in different behaviors during their visit such as less foraging and more resting behavior, a detail future studies should consider. Alternatively, injured or otherwise compromised animals could develop a profile-specific microbiome independent of their movement behavior. We believe these are exciting relationships which will need to be disentangled with future studies using high resolution tracking, longitudinal stress and health assessments, and microbiota sampling, as well as key events like vaccination and replenishment of litter that may drive these changes.

## Conclusion

Overall, our study highlights the potential of gut microbial profiles to act as biomarkers for behavioral traits and stress-related states in cage-free chickens. We believe that understanding the directionality of the complex behavior-microbiome interactions — whether the microbiome shapes behavior, vice versa, or both — is a promising avenue for future research.

Future work should adopt longitudinal approaches to capture temporal variation in microbiota, behavior, as well as health and stress related indicators. Such efforts could help determine whether behavioral traits, or their associated microbiota profiles, could serve as reliable indicators of welfare status, such as chronic stress, social dysfunction, or subclinical health conditions, in poultry production systems.

## Supporting information

S1 File DNA extraction and 16S rRNA gene sequencing protocol. (PDF)

S2 File Distribution across all pens of the 20% top and bottom animals. (TXT)

S3 File Analysis of pen: ANOVA results on alpha- and beta-diversity, and PERMANOVA results. (TXT)

S4 File Analysis of extreme litter visit behavior: ANOVA results on alpha- and beta-diversity, and PERMANOVA results. (TXT)

S5 File Analysis of other behavioral traits: ANOVA results on alpha- and beta-diversity, and PERMANOVA results. (TXT)

S1 Table Metadata table. (XLSX)

S2 Table ASV table. (TXT)

S3 Table Litter visit behavior analysis: DESeq2 results for differentially abundant ASVs, Genera, Families, and Orders between extreme groups. (XLSX)

## Abbreviations

ASV: Amplicon Sequence Variant
DA: Differentially Abundant
LIT: floor littered area
LIT-: Group of hens with fewer than 10 daily litter visits (bottom 20%)
LIT+: Group of hens with more than 30 daily litter visits (top 20%)
LLT: lower-level tier
MDL+: Group of hens with the higher values for mean duration litter (top 20%)
MDL-: Group of hens with the lower values for mean duration litter (bottom 20%)
MDW+: Group of hens with the higher values for mean duration wintergarden (top 20%)
MDW-: Group of hens with the lower values for mean duration wintergarden (bottom 20%)
NBT: Nest box tier
NMDS: Non-Metric Multidimensional Scaling
PERMANOVA: Permutational Multivariate Analysis of Variance
Rho: Rank correlation coefficient
SRA: Short-Read Archive
TLT: top-level tier
WG: wintergarden area
WOA: week of age
