# Supplementary material for: Exploring the relationships between the gut microbiome composition and movement patterns of laying hens in a multitier cage-free housing system
Source: PLoS One. 2026 Jan 9;21(1):e0340059. doi: 10.1371/journal.pone.0340059 (PMC12788645; doi:10.1371/journal.pone.0340059)
Supplement: S1 File — (PDF) [file pone.0340059.s001.pdf]

DNA extraction

A modified version of the protocol by Godon et al. (1997) was used for DNA extraction. For each animal, 200 mg of frozen fecal sample were resuspended with a mix of 250 µl of guanidine thiocyanate buffer (4 M guanidine thiocyanate–0.1 M Tris (pH 7.5) and 40 µl of 10% *N*-lauroyl sarcosine-–0.1 M phosphate buffer (pH 8.0)) and 500 µl of 5% *N*-lauroyl sarcosine; the tube was incubated at 70°C for 1 h. One volume (750 µl) of 0.1-mm-diameter silica beads (Sigma) was added, and tubes were shaken for 10 min at the maximum speed of a Vibrobroyeur MM200 (Retsch, Germany). Tubes were vortexed and centrifuged at 14000 rpm 5 min at 4°C. After recovery of the supernatant, 30 µl of Proteinase K (Chemagic STAR DNA BTS kit, Perkin Elmer, USA) were added and samples were incubated for 10 min at 70°C at 250 rpm in Multi-Therm (Benchmark Scientific, USA), then for 5 min at 95°C for enzyme inactivation. Tubes were centrifuged at 14000 rpm for 5 min at 4°C and supernatant was transferred in a deepwell. The plate was transferred on the nucleic acid workstation Chemagic STAR (Hamilton, Perkin Elmer, USA) and the extraction protocol was performed with Chemagic STAR DNA BTS kit (Perkin Elmer, USA) according to the manufacturer's instructions.

Primer design and library preparation

The V3-V4 hyper-variable regions of the 16S rDNA gene were amplified from the DNA extracts during the first PCR step using universal primers PCR1F_343 and PCR1_R784 (Table 1) which are fusion primers as described by Nadkarni et al. (2002). This PCR was performed using 2 U of a DNA-free Taq DNA Polymerase and 1x Taq DNA polymerase buffer (MTP Taq DNA Polymerase, Sigma-Aldrich, USA). The buffer was completed with 10 nmol of dNTP mixture (Sigma-Aldrich, USA), 15 nmol of each primer (Eurofins) and Nuclease-free water (Qiagen, Germany) in a final volume of 50 µl.

The PCR reaction was carried out in a T100 Thermal cycler (Biorad, USA) as follows: an initial denaturation step (94°C 10 min) was followed by 30 cycles of amplification (94°C for 1 min, 68° C for 1 min and 72°c for 1 min) and a final elongation step at 72°C for 10 min. Amplicons were then purified using a magnetic beads CleanPCR (Clean NA, GC biotech B.V.,The Nederlands) in a 96 well format. The concentration of the purified amplicons was controlled using a Nanodrop spectrophotometer (Thermo Scientific, USA) and a subset of amplicons size was controlled on a Fragment Analyzer (AATI, USA) with the reagent kit ADNdb 910 (35-1,500 bp).

Sample multiplexing was performed by adding tailor-made 6 bp unique indexes during the second PCR step at the same time as the second part of the P5/P7 adapters to obtain primers PCR2_P7F and reverse primer PCR2_P7R(Table 1). This second PCR step was performed on 50–200 ng of purified amplicons from the first PCR using 2.5 U of a DNA free Taq DNA Polymerase and 1xTaq DNA polymerase buffer. The buffer was completed with 10 nmol of dNTP mixture (Sigma-Aldrich, USA), 25 nmol of each primer (Eurofins, Luxembourg) and Nuclease-free water (Qiagen, Germany) up to a final volume of 50 µl. The PCR reaction was carried out on a T100 Thermal cycler with an initial denaturation step (94°C for 10 min), 12 cycles of amplification (94°C for 1 min, 65°C for 1 min and 72°C for 1 min) and a final elongation step at 72°C for 10 min. Amplicons were purified as described for the first PCR reaction. The concentration of the purified amplicons was measured using Nanodrop spectrophotometer (Thermo Scientific, USA) and the quality of a subset of amplicons (12 samples per sequencing run) was controlled on a Fragment Analyzer (AATI, USA) with the reagent kit ADNdb 910 (35-1,500 bp).

Controls were carried out to ensure that the high number of PCR cycles (35 cycles for PCR 1 + 12 cycles for PCR2) did not create significant amounts of PCR chimera or other artifacts. The region of the 16S rDNA gene to be sequenced has a length of 467 bp for a total amplicon length of 522 bp after PCR 1 and of 588 bp after PCR 2 (using the 16S rDNA gene of E. coli as a reference).

Negative controls to assess technical background were included using Nuclease-free water (Qiagen, Germany) in place of the extracted DNA during the library preparation.

All libraries were pooled with equal amounts in order to generate equivalent number of raw reads for each library. The DNA concentration of the pool (no dilution, diluted 10x and 25x in EB + Tween 0.5% buffer) was quantified on a Qubit Fluorometer (Thermofisher Scientific, USA). The pool, at a final concentration between 5 and 20 nM, was used for sequencing.

Sequencing

The pool was denatured (NaOH 0.1N) and diluted to 7 pM. The PhiX Control v3 (Illumina, USA) was added to the pool at 15% of the final concentration as described in the Illumina procedure. 600 µl of this pool and PhiX mixture were loaded onto the Illumina MiSeq cartridge according to the manufacturer's instructions using MiSeq Reagent Kit v3 (2x300 bp Paired-End Reads, 15 Gb output). FastQ files were generated at the end of the run (MiSeq Reporter software, Illumina, USA) to perform the quality control. The quality of the run was checked internally using PhiX Control and then each paired-end sequence was assigned to its sample using the multiplexing index.

References:

Godon JJ, Zumstein E, Dabert P, et al. Molecular microbial diversity of an anaerobic digestor as determined by small-subunit rDNA sequence analysis. Appl Environ Microbiol. 1997;63:2802–13

Nadkarni MA, Martin FE, Jacques NA, Hunter N. Determination of bacterial load by real-time PCR using a broad-range universal) probe and primers set. Microbiology. 2002; 148: 257–66.

Lluch, J., Servant, F., Paisse, S., Valle, C., Valiere, S., Kuchly, C., et al. (2015). The Characterization of novel tissue microbiota using an optimized 16S metagenomic sequencing pipeline. PLoS ONE 10:e0142334. doi:0.1371/journal.pone.0142334

Table 1

Name	Sequence
PCR1F_343	CTTTCCCTACACGACGCTCTTCCGATCT-ACGGRAGGCAGCAG<br>partial P5 adapter–primer
PCR1_R784	GGAGTTCAGACGTGTGCTCTTCCGATCTTACCAGGGTATCTAATCCT<br>partial P7 adapter–primer
PCR2_P5F	AATGATACGGCGACCACCGAGATCTACACT-CTTTCCCTACACGAC<br>partial P5 adapter–primer targeting primer 1F
PCR2_P7R	CAAGCAGAAGACGGCATACGAGAT-NNNNNN-GTGACT-GGAGTTCAGACGTGT<br>partial P7 adapter including index–primer targeting primer 1R

Primers PCR1F_343 and PCR1_R784 are specific for the 16S rDNA gene of 95% of the bacteria in the Ribosomal Database Project and part of the P5/P7 adapter targeted by the second PCR step.
